# Supplementary figures and images for: ZBTB48 is a priming factor regulating B-cell-specific CIITA expression
Source: EMBO J. 2024 Nov 19;43(24):2. doi: 10.1038/s44318-024-00306-y (PMC11649694; doi:10.1038/s44318-024-00306-y)

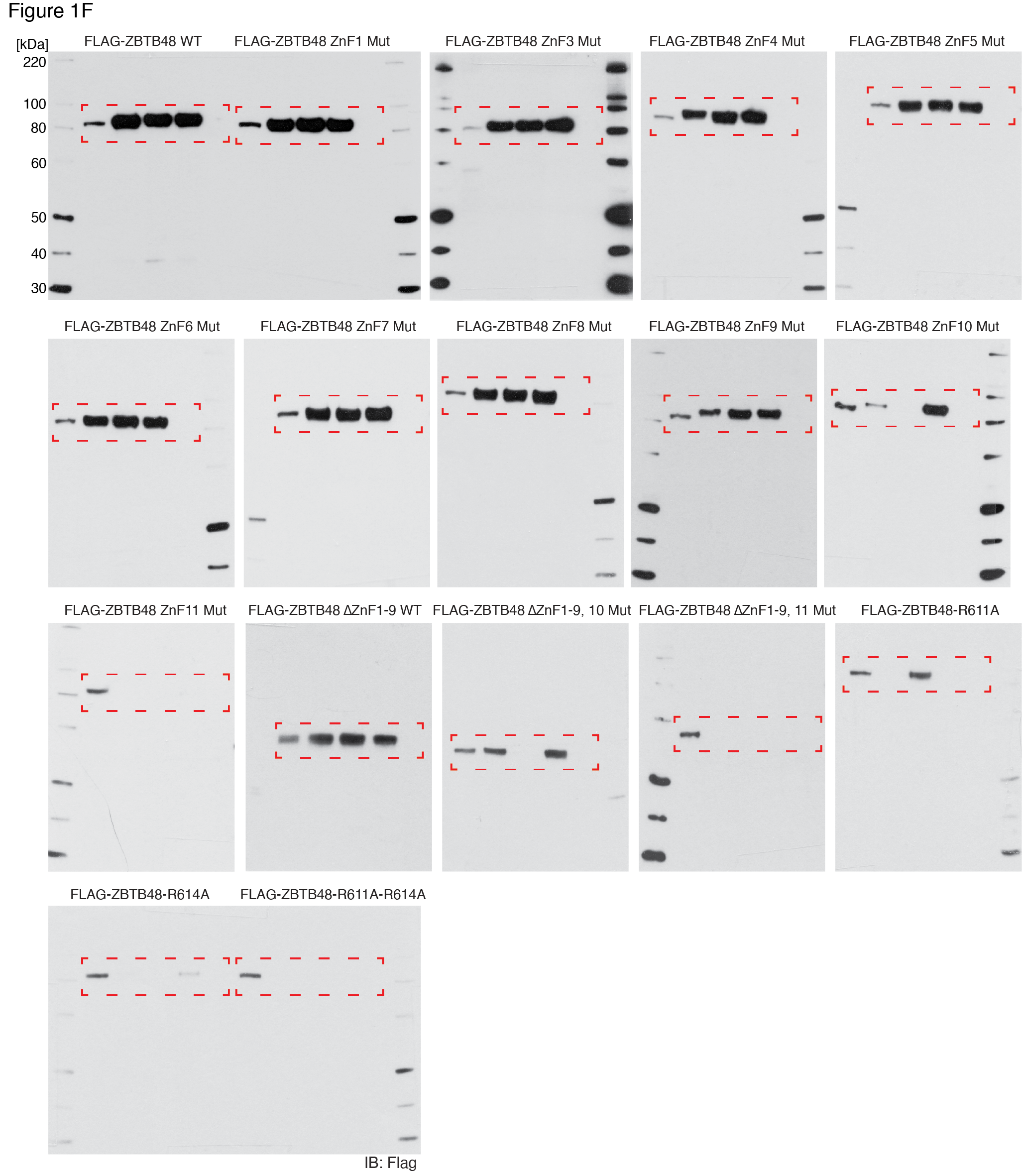

Supplement: Supplementary file 8 — Source data Fig. 1 [file 44318_2024_306_MOESM8_ESM.zip › Figure 1/1F/Western Blot for DNA pulldown assay.png]

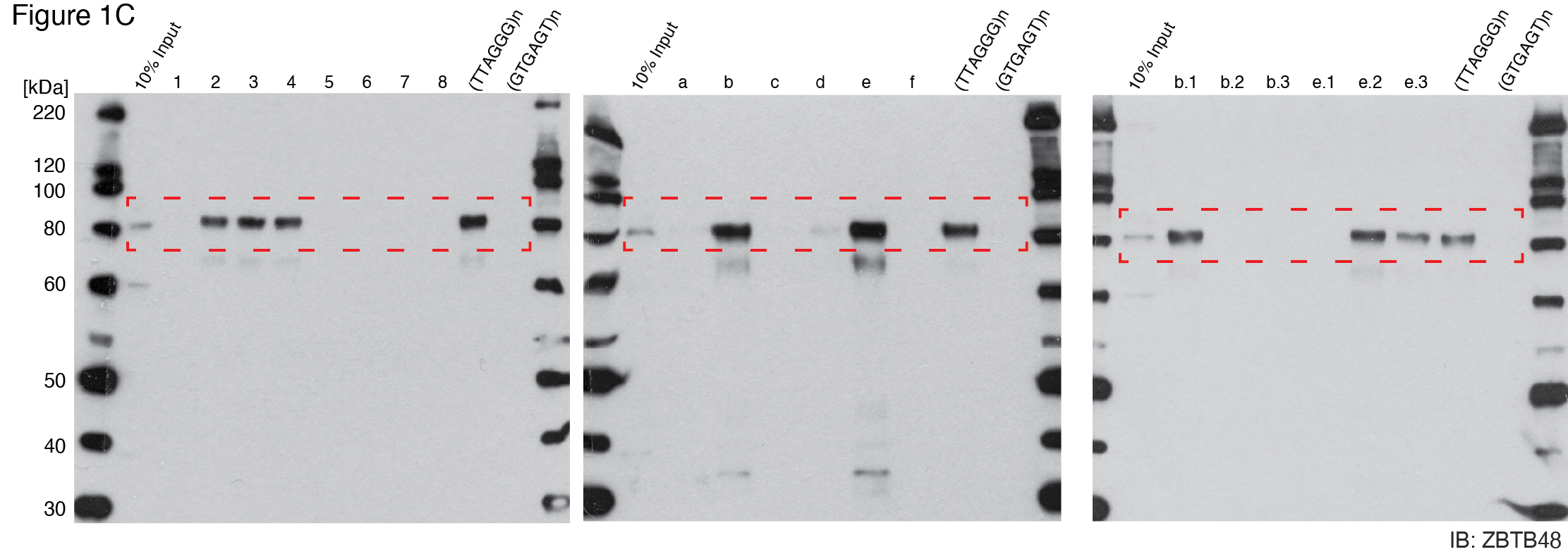

Supplement: Supplementary file 8 — Source data Fig. 1 [file 44318_2024_306_MOESM8_ESM.zip › Figure 1/1C/Western Blot for pulldown assay.png]

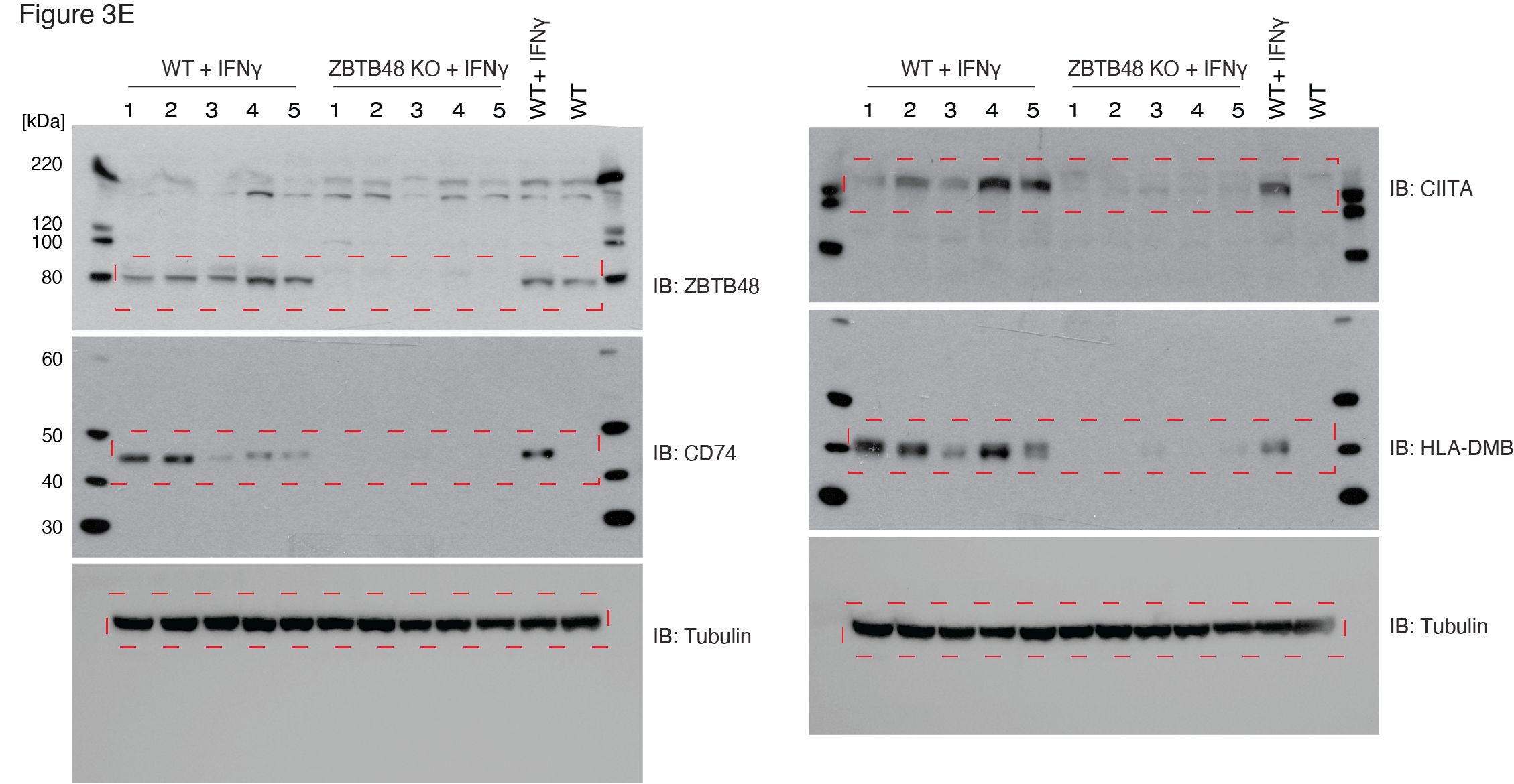

Supplement: Supplementary file 9 — Source data Fig. 3 [file 44318_2024_306_MOESM9_ESM.zip › Figure 3/3E/Western blot_U2OS WT, KO clones_IFNg.png]

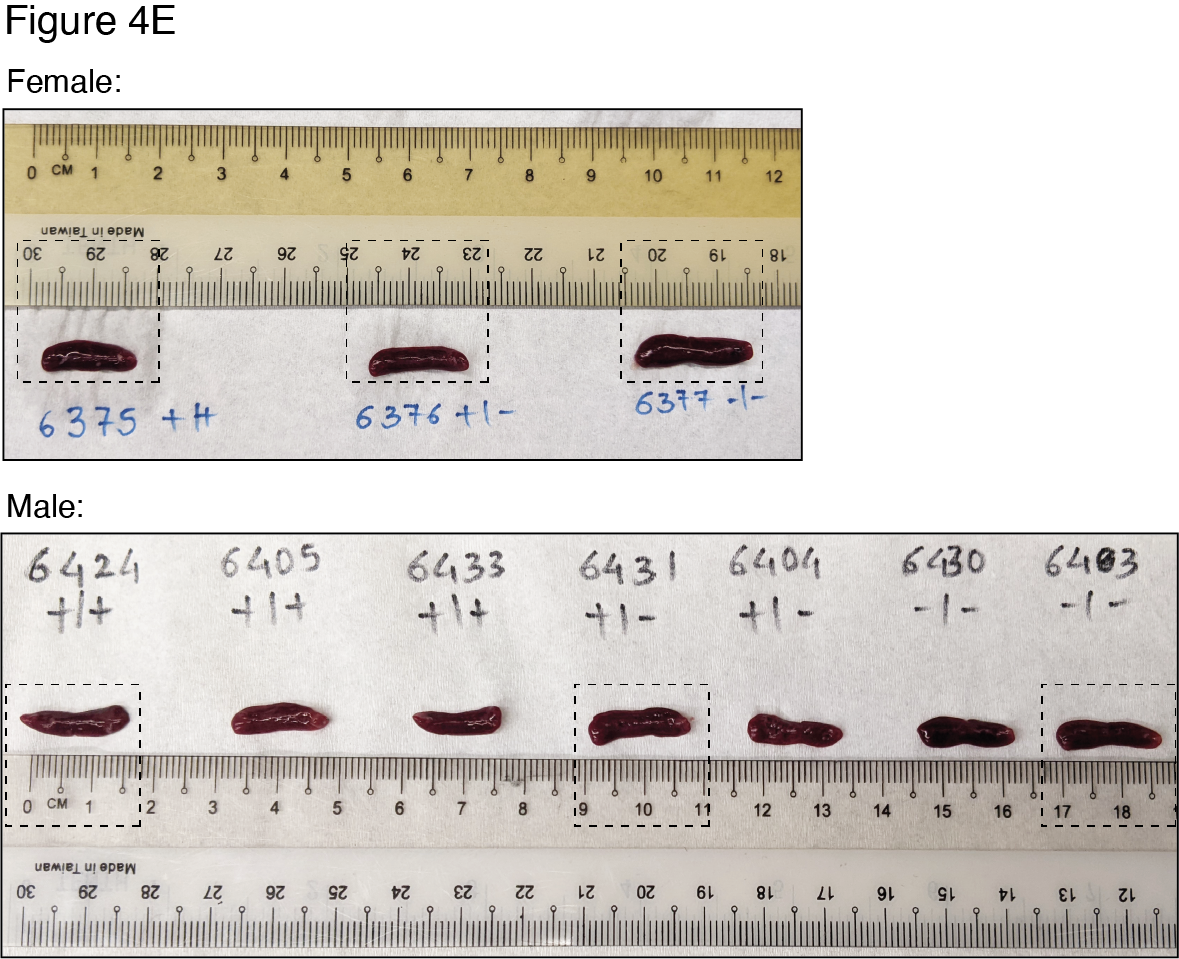

Supplement: Supplementary file 10 — Source data Fig. 4 [file 44318_2024_306_MOESM10_ESM.zip › Figure 4/4E/Spleen images_WT,Het,KO mice.png]

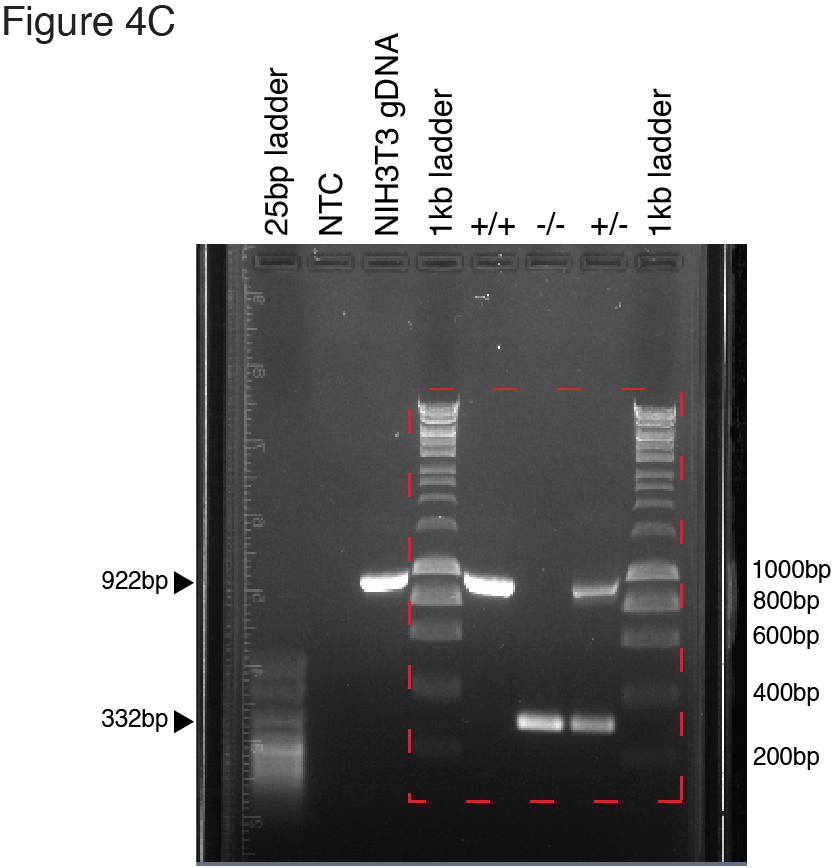

Supplement: Supplementary file 10 — Source data Fig. 4 [file 44318_2024_306_MOESM10_ESM.zip › Figure 4/4C/Electrophoresis gel_WT,Het,KO mice.png]

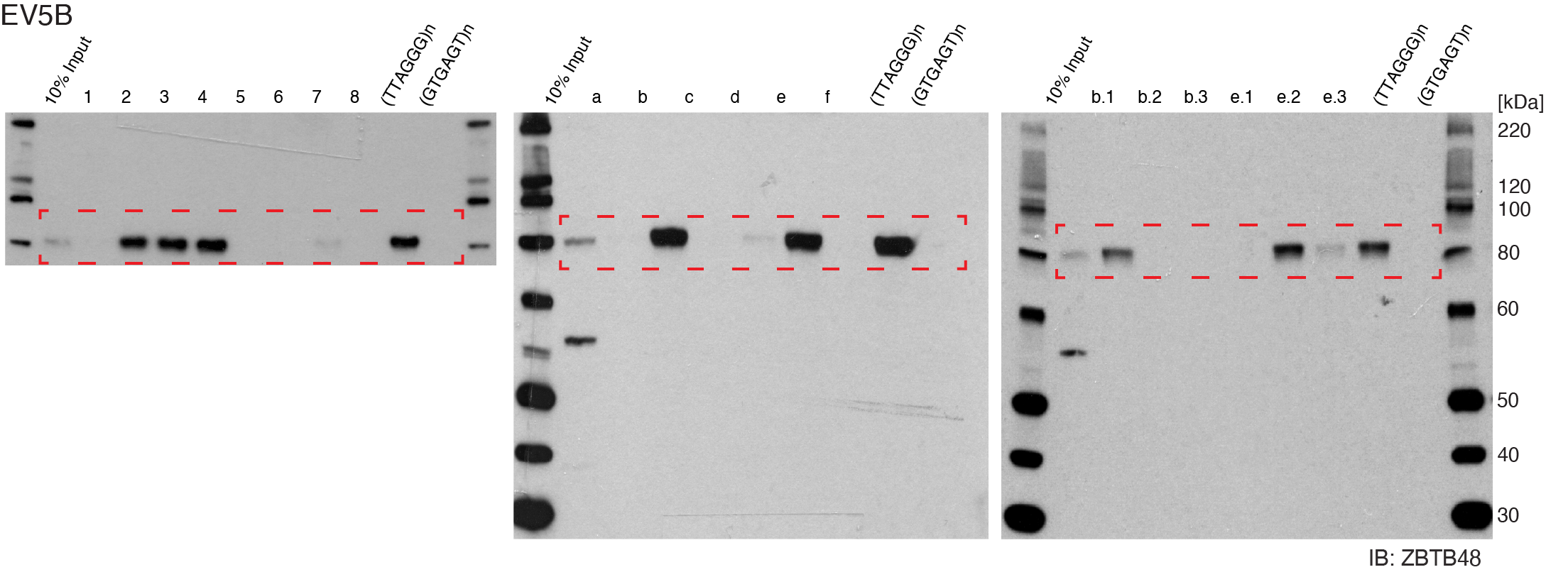

Supplement: Supplementary file 13 — Figure EV5 Source Data [file 44318_2024_306_MOESM13_ESM.zip › Figure EV5/EV5B/Western blot for DNA pulldown assay_EV5B.png]
