# Supplementary material for: ZBTB48 is a priming factor regulating B-cell-specific CIITA expression
Source: EMBO J. 2024 Nov 19;43(24):2. doi: 10.1038/s44318-024-00306-y (PMC11649694; doi:10.1038/s44318-024-00306-y)
Supplement: Supplementary file 14 — Expanded View Figures [file 44318_2024_306_MOESM14_ESM.pdf]

Expanded View Figures

Figure EV1. ZBTB48 regulates IFNγ-induced CIITA pIII expression.

(**A**) ChIP-seq tracks depicting ZBTB48 binding peaks at CIITA pIII in HeLa cells but not in the KO cells ($n = 4$). No reads are observed in the corresponding RNA-seq tracks in these uninduced cells ($n = 5$; data re-analyzed from Jahn et al (Jahn et al, 2017)). (**B**) Relative mRNA expression of total (pan-)CIITA and the three promoter-specific transcripts in each five independent HeLa WT and ZBTB48 KO clones treated with or without 250 U/ml IFNγ for 24 h. Data represents mean ± SEM. Data points with gray circles are imputed due to lack of detectable Ct values. The $\log_2$ fold change is calculated relative to the average of five untreated WT clones. p values were calculated by 2-way ANOVA with Sidak correction for multiple comparisons ($n = 5$); ***$p < 0.001$. pan-CIITA: WT vs WT + IFNγ: $p < 1E\text{-}15$, KO vs KO + IFNγ: $p < 1E\text{-}15$, WT vs KO: $p = 1.000$, WT + IFNγ vs KO + IFNγ: $p = 0.941$; CIITA pIII: WT vs WT + IFNγ: $p < 1E\text{-}15$, KO vs KO + IFNγ: $p = 5.386E\text{-}12$, WT vs KO: $p = 0.999$, WT + IFNγ vs KO + IFNγ: $p < 1E\text{-}15$; CIITA pIV: WT vs WT + IFNγ: $p = 2.260E\text{-}13$, KO vs KO + IFNγ: $p = 2.965E\text{-}11$, WT vs KO: $p = 0.712$, WT + IFNγ vs KO + IFNγ: $p = 0.077$. (**C**) Differential expression analysis of the RNA-seq quantification comparing 5 U2OS WT clones +/− IFNγ treatment (250 U/ml IFNγ for 24 h). Genes belonging to the CIITA-MHC-II family are in blue and the rest of the IFNγ response genes (ISGs) in yellow. Additional proteins that expressed above a two-dimensinal cut-off of >32-fold enrichment (owing to the large number of strongly induced genes) and $p < 0.01$ are in salmon. (**D**) Differential expression analysis of the RNA-seq quantification comparing 5 U2OS ZBTB48 KO clones +/− IFNγ treatment (250 U/ml IFNγ for 24 h) depicted as in (**C**). (**E**) GO term analysis for enriched genes in pair-wise comparisons in Fig. 3D, Fig. EV1C,D, and Fig. EV1G depicting significantly enriched GO terms. The size of the dots corresponds to the number of genes representing each term. (**F**) Heatmap of differentially expressed MHC-II family genes detected in RNA-seq data comparing 5 U2OS WT and ZBTB48 KO clones +/− IFNγ. (**G**) Differential expression analysis of the RNA-seq quantification comparing 5 U2OS WT and ZBTB48 KO clones (without IFNγ treatment). Genes are colored as in (**C**, **D**), but here a >twofold enrichment cut-off was applied (as in Fig. 3D) given that baseline expression differences are compared.

 © The Author(s)

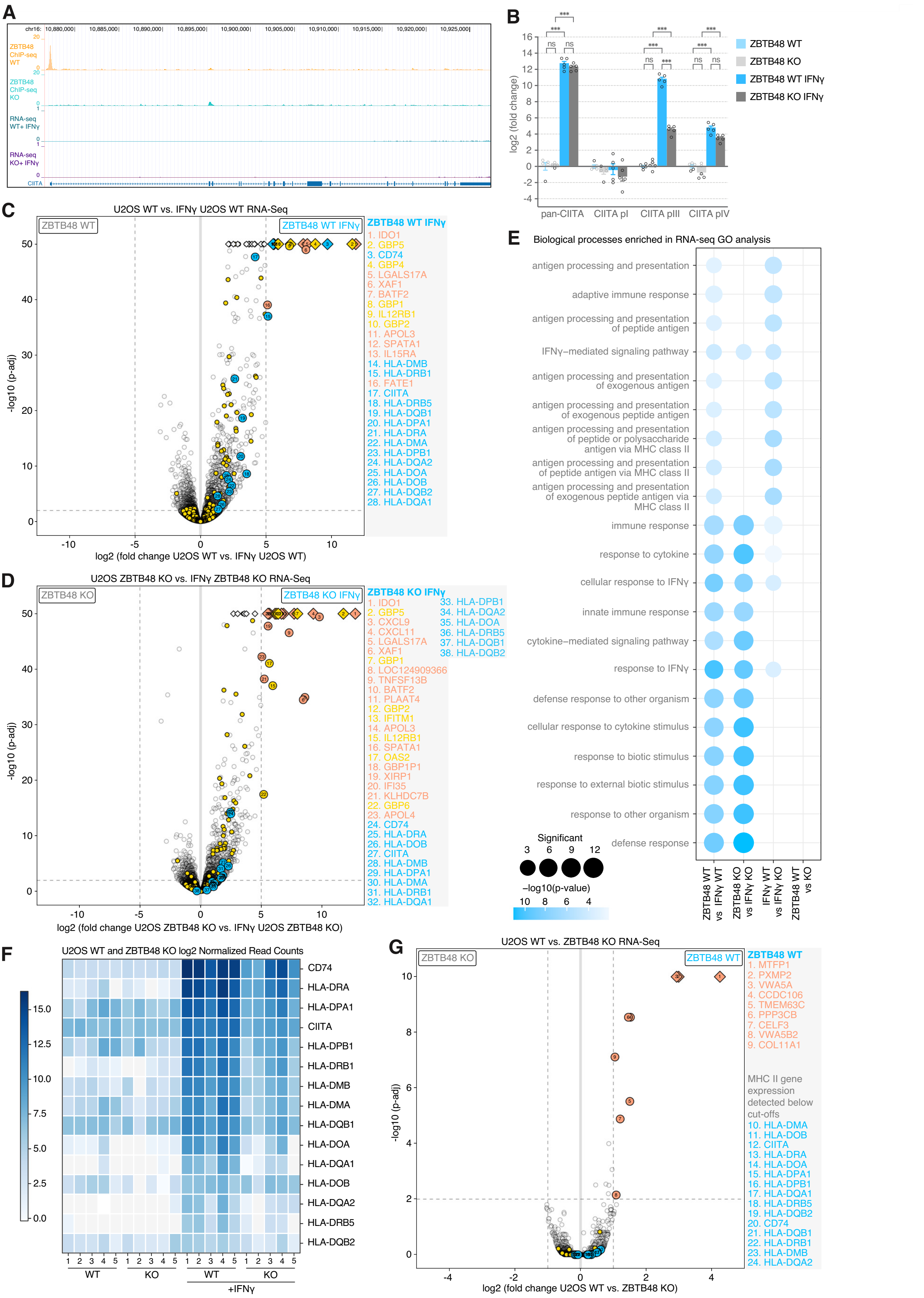
A
ZBTB48 ChIP-seq WT
ZBTB48 ChIP-seq KO
RNA-seq WT+ IFNγ
RNA-seq KO+ IFNγ
CIITA
B
log2 (fold change)
ZBTB48 WT
ZBTB48 KO
ZBTB48 WT IFNγ
ZBTB48 KO IFNγ
pan-CIITA
CIITA pI
CIITA pIII
CIITA pIV
C
U2OS WT vs. IFNγ U2OS WT RNA-Seq
ZBTB48 WT
ZBTB48 WT IFNγ
-log10 (p-adj)
log2 (fold change U2OS WT vs. IFNγ U2OS WT)
ZBTB48 WT IFNγ
1. IDO1
2. GBP5
3. CD74
4. GBP4
5. LGALS17A
6. XAF1
7. BATF2
8. GBP1
9. IL12RB1
10. GBP2
11. APOL3
12. SPATA1
13. IL15RA
14. HLA-DMB
15. HLA-DRB1
16. FATE1
17. CIITA
18. HLA-DRB5
19. HLA-DQB1
20. HLA-DPA1
21. HLA-DRA
22. HLA-DMA
23. HLA-DPB1
24. HLA-DQA2
25. HLA-DOA
26. HLA-DOB
27. HLA-DQB2
28. HLA-DQA1
D
U2OS ZBTB48 KO vs. IFNγ ZBTB48 KO RNA-Seq
ZBTB48 KO
ZBTB48 KO IFNγ
-log10 (p-adj)
log2 (fold change U2OS ZBTB48 KO vs. IFNγ U2OS ZBTB48 KO)
ZBTB48 KO IFNγ
1. IDO1
2. GBP5
3. CXCL9
4. CXCL11
5. LGALS17A
6. XAF1
7. GBP1
8. LOC124909366
9. TNFSF13B
10. BATF2
11. PLAAT4
12. GBP2
13. IFITM1
14. APOL3
15. IL12RB1
16. SPATA1
17. OAS2
18. GBP1P1
19. XIRP1
20. IFI35
21. KLHDC7B
22. GBP6
23. APOL4
24. CD74
25. HLA-DRA
26. HLA-DOB
27. CIITA
28. HLA-DMB
29. HLA-DPA1
30. HLA-DMA
31. HLA-DRB1
32. HLA-DQA1
33. HLA-DPB1
34. HLA-DQA2
35. HLA-DOA
36. HLA-DRB5
37. HLA-DQB1
38. HLA-DQB2
E
Biological processes enriched in RNA-seq GO analysis
antigen processing and presentation
adaptive immune response
antigen processing and presentation of peptide antigen
IFNγ−mediated signaling pathway
antigen processing and presentation of exogenous antigen
antigen processing and presentation of exogenous peptide antigen
antigen processing and presentation of peptide or polysaccharide antigen via MHC class II
antigen processing and presentation of peptide antigen via MHC class II
antigen processing and presentation of exogenous peptide antigen via MHC class II
immune response
response to cytokine
cellular response to IFNγ
innate immune response
cytokine−mediated signaling pathway
response to IFNγ
defense response to other organism
cellular response to cytokine stimulus
response to biotic stimulus
response to external biotic stimulus
response to other organism
defense response
Significant
−log10(p-value)
ZBTB48 WT vs IFNγ WT
ZBTB48 KO vs IFNγ KO
IFNγ WT vs IFNγ KO
ZBTB48 WT vs KO
F
U2OS WT and ZBTB48 KO log2 Normalized Read Counts
CD74
HLA-DRA
HLA-DPA1
CIITA
HLA-DPB1
HLA-DRB1
HLA-DMB
HLA-DMA
HLA-DQB1
HLA-DOA
HLA-DQA1
HLA-DOB
HLA-DQA2
HLA-DRB5
HLA-DQB2
WT
KO
+IFNγ
G
U2OS WT vs. ZBTB48 KO RNA-Seq
ZBTB48 KO
ZBTB48 WT
-log10 (p-adj)
log2 (fold change U2OS WT vs. ZBTB48 KO)
ZBTB48 WT
1. MTFP1
2. PXMP2
3. VWA5A
4. CCDC106
5. TMEM63C
6. PPP3CB
7. CELF3
8. VWA5B2
9. COL11A1
MHC II gene expression detected below cut-offs
10. HLA-DMA
11. HLA-DOB
12. CIITA
13. HLA-DRA
14. HLA-DOA
15. HLA-DPA1
16. HLA-DPB1
17. HLA-DQA1
18. HLA-DRB5
19. HLA-DQB2
20. CD74
21. HLA-DQB1
22. HLA-DRB1
23. HLA-DMB
24. HLA-DQA2

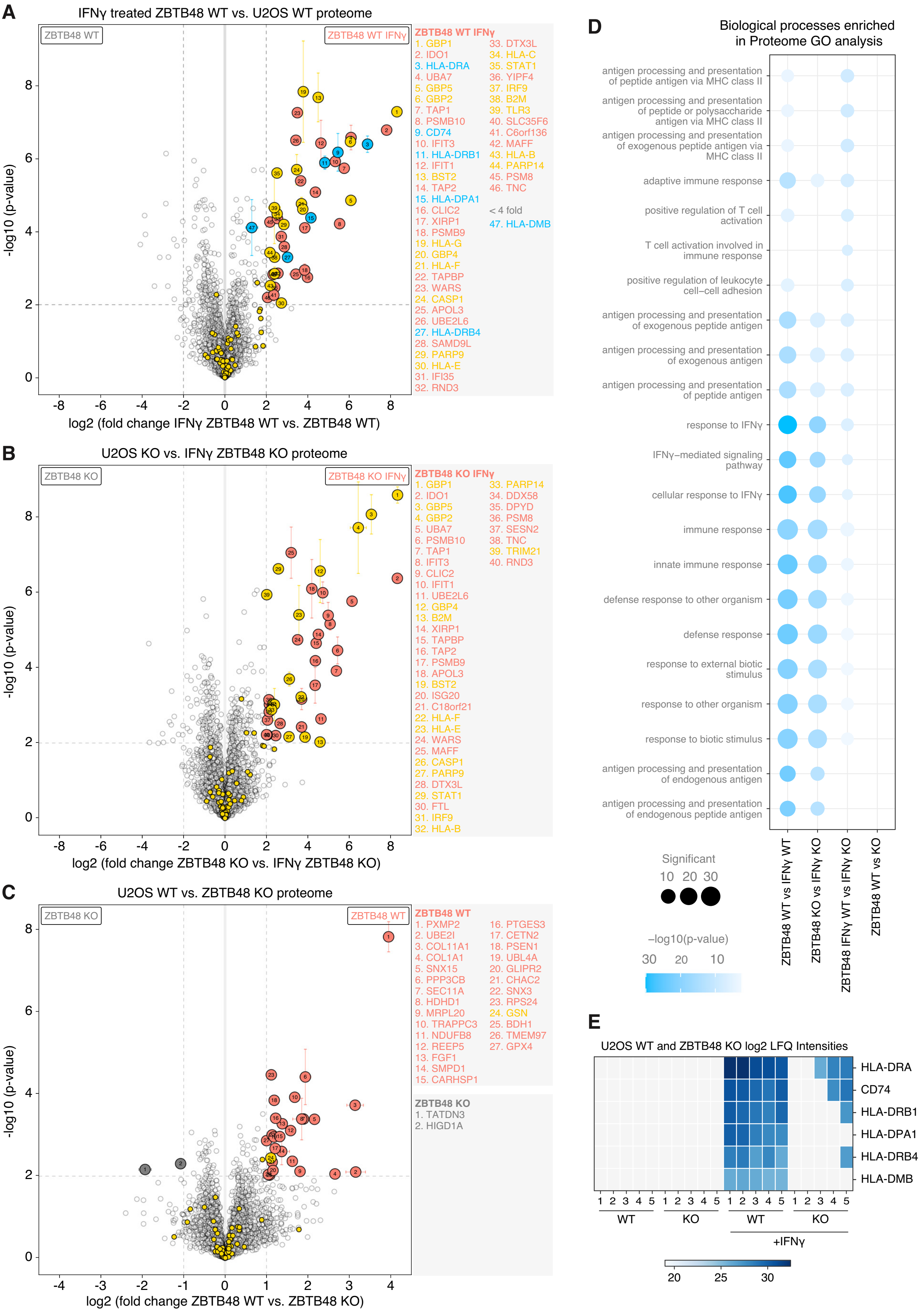
A
IFNγ treated ZBTB48 WT vs. U2OS WT proteome
ZBTB48 WT
ZBTB48 WT IFNγ
-log10 (p-value)
log2 (fold change IFNγ ZBTB48 WT vs. ZBTB48 WT)
ZBTB48 WT IFNγ
1. GBP1
2. IDO1
3. HLA-DRA
4. UBA7
5. GBP5
6. GBP2
7. TAP1
8. PSMB10
9. CD74
10. IFIT3
11. HLA-DRB1
12. IFIT1
13. BST2
14. TAP2
15. HLA-DPA1
16. CLIC2
17. XIRP1
18. PSMB9
19. HLA-G
20. GBP4
21. HLA-F
22. TAPBP
23. WARS
24. CASP1
25. APOL3
26. UBE2L6
27. HLA-DRB4
28. SAMD9L
29. PARP9
30. HLA-E
31. IFI35
32. RND3
33. DTX3L
34. HLA-C
35. STAT1
36. YIPF4
37. IRF9
38. B2M
39. TLR3
40. SLC35F6
41. C6orf136
42. MAFF
43. HLA-B
44. PARP14
45. PSM8
46. TNC
< 4 fold
47. HLA-DMB
B
U2OS KO vs. IFNγ ZBTB48 KO proteome
ZBTB48 KO
ZBTB48 KO IFNγ
-log10 (p-value)
log2 (fold change ZBTB48 KO vs. IFNγ ZBTB48 KO)
ZBTB48 KO IFNγ
1. GBP1
2. IDO1
3. GBP5
4. GBP2
5. UBA7
6. PSMB10
7. TAP1
8. IFIT3
9. CLIC2
10. IFIT1
11. UBE2L6
12. GBP4
13. B2M
14. XIRP1
15. TAPBP
16. TAP2
17. PSMB9
18. APOL3
19. BST2
20. ISG20
21. C18orf21
22. HLA-F
23. HLA-E
24. WARS
25. MAFF
26. CASP1
27. PARP9
28. DTX3L
29. STAT1
30. FTL
31. IRF9
32. HLA-B
33. PARP14
34. DDX58
35. DPYD
36. PSM8
37. SESN2
38. TNC
39. TRIM21
40. RND3
C
U2OS WT vs. ZBTB48 KO proteome
ZBTB48 KO
ZBTB48 WT
-log10 (p-value)
log2 (fold change ZBTB48 WT vs. ZBTB48 KO)
ZBTB48 WT
1. PXMP2
2. UBE2I
3. COL11A1
4. COL1A1
5. SNX15
6. PPP3CB
7. SEC11A
8. HDHD1
9. MRPL20
10. TRAPPC3
11. NDUFB8
12. REEP5
13. FGF1
14. SMPD1
15. CARHSP1
16. PTGES3
17. CETN2
18. PSEN1
19. UBL4A
20. GLIPR2
21. CHAC2
22. SNX3
23. RPS24
24. GSN
25. BDH1
26. TMEM97
27. GPX4
ZBTB48 KO
1. TATDN3
2. HIGD1A
D
Biological processes enriched
in Proteome GO analysis
antigen processing and presentation of peptide antigen via MHC class II
antigen processing and presentation of peptide or polysaccharide antigen via MHC class II
antigen processing and presentation of exogenous peptide antigen via MHC class II
adaptive immune response
positive regulation of T cell activation
T cell activation involved in immune response
positive regulation of leukocyte cell−cell adhesion
antigen processing and presentation of exogenous peptide antigen
antigen processing and presentation of exogenous antigen
antigen processing and presentation of peptide antigen
response to IFNγ
IFNγ−mediated signaling pathway
cellular response to IFNγ
immune response
innate immune response
defense response to other organism
defense response
response to external biotic stimulus
response to other organism
response to biotic stimulus
antigen processing and presentation of endogenous antigen
antigen processing and presentation of endogenous peptide antigen
ZBTB48 WT vs IFNγ WT
ZBTB48 KO vs IFNγ KO
ZBTB48 IFNγ WT vs IFNγ KO
ZBTB48 WT vs KO
Significant
10 20 30
−log10(p-value)
30 20 10
E
U2OS WT and ZBTB48 KO log2 LFQ Intensities
HLA-DRA
CD74
HLA-DRB1
HLA-DPA1
HLA-DRB4
HLA-DMB
1 2 3 4 5 1 2 3 4 5 1 2 3 4 5 1 2 3 4 5
WT
KO
WT
KO
+IFNγ
20 25 30

 © The Author(s)

Figure EV2. ZBTB48 exclusively affects the CIITA-MHC-II expression program upon IFNγ stimulation at the protein level.

(**A**) Differential expression analysis of label-free quantitative protein expression comparing 5 U2OS WT clones +/− IFNγ treatment (250 U/ml IFNγ for 48 h). Genes belonging to the CIITA-MHC-II family are in blue and the rest of the IFNγ response genes (ISGs) in yellow. Additional proteins that expressed above a two-dimensinal cut-off of >4-fold enrichment (owing to the large number of strongly induced genes) and $p < 0.01$ are in salmon. (**B**) Differential expression analysis of label-free quantitative protein expression comparing 5 U2OS ZBTB48 KO clones +/− IFNγ treatment (250 U/ml IFNγ for 48 h) depicted as in (**A**). (**C**) Differential expression analysis of label-free quantitative protein expression comparing 5 U2OS WT and ZBTB48 KO clones (without IFNγ treatment). Genes are colored as in (**A**, **B**), but here a > 2-fold enrichment cut-off was applied (as in Fig. 3D) given that baseline expression differences are compared. (**A–C**) Two-dimensional error bars represent the standard deviation based on iterative imputation cycles during the label-free analysis to substitute missing values (e.g., no detection in the KO clones) and the measure of center is mean. *p* values were calculated by independent sample *t* test ($n = 5$). (**D**) GO term analysis for enriched proteins in pair-wise comparisons in Figs. 3F and EV2A–C depicting significantly enriched GO terms. The size of the dots corresponds to the number of genes representing each term. (**E**) Heatmap of differentially expressed MHC-II family genes detected in the proteome data comparing 5 U2OS WT and ZBTB48 KO clones +/− IFNγ.

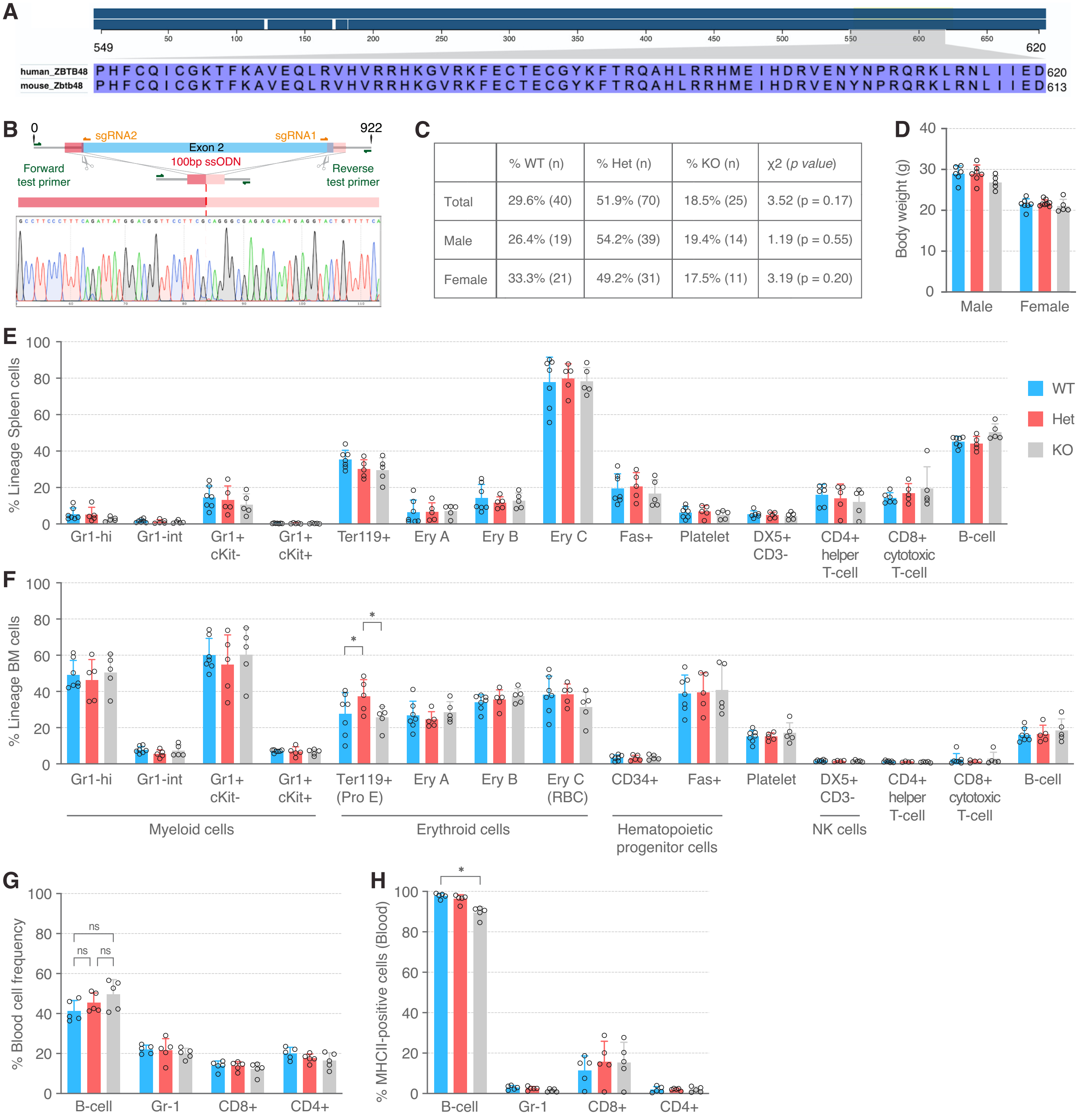

	% WT (n)	% Het (n)	% KO (n)	χ2 (*p value*)
Total	29.6% (40)	51.9% (70)	18.5% (25)	3.52 (p = 0.17)
Male	26.4% (19)	54.2% (39)	19.4% (14)	1.19 (p = 0.55)
Female	33.3% (21)	49.2% (31)	17.5% (11)	3.19 (p = 0.20)

Figure EV3. Loss of ZBTB48 does not affect abundance of blood cell populations.

(**A**) Protein sequence alignment using Clustal Omega of human and mouse ZBTB48 (top) with a zoom in to the DNA-binding region including ZnF10, ZnF11 and the C-terminal arm that are required for binding to CIITA pIII (bottom). (**B**) Schematic of the sgRNA and ssODN design used for creation of the ZBTB48 KO strain (top) and Sanger sequencing verification of successful and precise deletion (bottom) in ZBTB48$^{-/-}$ animals. (**C**) χ^2-test comparing experimentally determined off-spring frequency in crosses between ZBTB48$^{+/-}$ animals. The genotype distribution follows Mendelian inheritance patterns for the total cohort (top), male (middle) and female (bottom) mice. (**D**) Body weight in g calculated separately for males and females across the three genotypes. Data represents mean ± SD (male: WT $n = 6$, Het $n = 7$, KO $n = 5$; female: WT $n = 7$, Het $n = 8$, KO $n = 5$). These data were used to generate the ratio with the spleen weight in Fig. 4D. (**E**) Percentage of spleen lineage cells in WT ($n = 7$), Het ($n = 5$) and KO ($n = 5$) mice. (**F**) Percentage of bone marrow lineage cells in WT ($n = 7$), Het ($n = 5$) and KO ($n = 5$) mice. (**G**) Percentage of blood cell frequencies in WT ($n = 7$), Het ($n = 5$) and KO ($n = 5$) mice. (**H**) Percentage of MHC-II-positive cells in the blood cell types from Fig. EV4F. For (**E–H**) data represents mean ± SD. p values were calculated by two-way ANOVA with Sidak correction for multiple comparisons; *$p < 0.05$. p values for the percentage cells in (**F**): WT vs Het: $p = 0.045$, Het vs KO: $p = 0.021$; in (H): WT vs KO: $p = 0.03$. Gr1-hi, Gr1 high; Gr1-int, Gr1 intermediate.

© The Author(s)

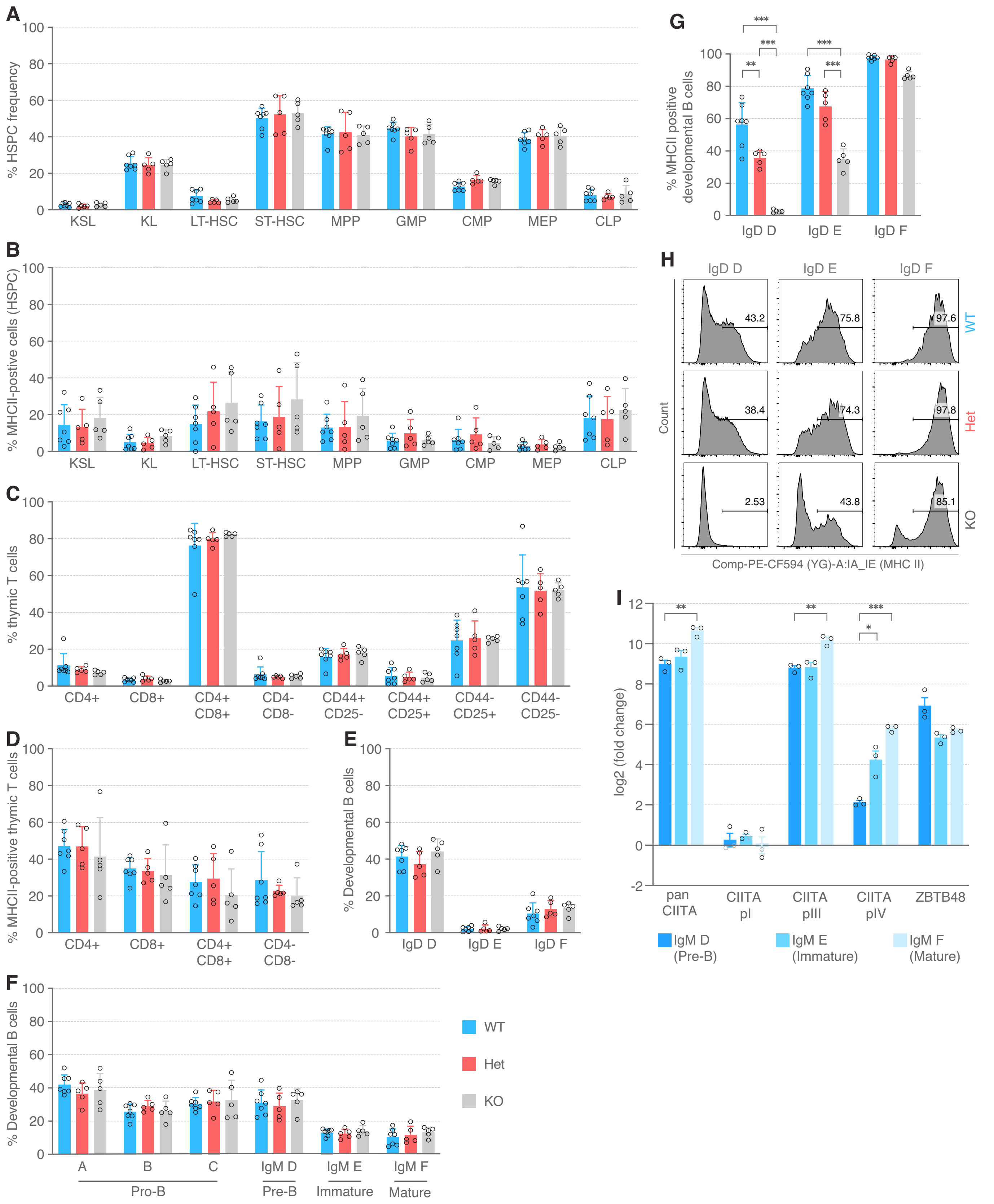
A
% HSPC frequency
KSL
KL
LT-HSC
ST-HSC
MPP
GMP
CMP
MEP
CLP
B
% MHCII-positive cells (HSPC)
C
% thymic T cells
CD4+
CD8+
CD4+ CD8+
CD4- CD8-
CD44+ CD25-
CD44+ CD25+
CD44- CD25+
CD44- CD25-
D
% MHCII-positive thymic T cells
E
% Developmental B cells
IgD D
IgD E
IgD F
F
A
B
C
Pro-B
IgM D
Pre-B
IgM E
Immature
IgM F
Mature
WT
Het
KO
G
% MHCII positive developmental B cells
H
Count
43.2
75.8
97.6
38.4
74.3
97.8
2.53
43.8
85.1
Comp-PE-CF594 (YG)-A:IA_IE (MHC II)
I
log2 (fold change)
pan CIITA
CIITA pI
CIITA pIII
CIITA pIV
ZBTB48
IgM D (Pre-B)
IgM E (Immature)
IgM F (Mature)

Figure EV4. Loss of ZBTB48 does not affect abundance of hematopoietic stem cells, developmental B cells and thymic T cells.

(**A**) Percentage of hematopoietic stem and progenitor cell (HSPC) types in WT ($n = 7$), Het ($n = 5$) and KO ($n = 5$) mice. KSL: c-kit$^+$ Sca-1$^+$ Lineage$^-$ (Lin$^-$), KL: c-kit$^+$ Lin$^-$, LT-HSC: long-term 'quiescent' hematopoietic stem cells, ST-HSC: short-term 'cycling' hematopoietic stem cells, MPP: multipotent progenitor (KSL CD34$^+$ flt3$^+$), GMP: granulocyte-macrophage progenitor, CMP: common myeloid progenitor, MEP: megakaryocyte-erythroid progenitor, CLP: common lymphoid progenitor. (**B**) Percentage of MHC-II-positive cells in HSPCs from Fig. EV4A. (**C**) Percentage of thymic T cells in WT ($n = 7$), Het ($n = 5$) and KO ($n = 5$) mice. (**D**) Percentage of MHCII-positive cells in thymic T cells from Fig. EV4C. (**E**) Percentage of development B cell types in WT ($n = 7$), Het ($n = 5$) and KO ($n = 5$) mice when gated by IgD. (**F**) Percentage of development B cell types in WT ($n = 7$), Het ($n = 5$) and KO ($n = 5$) mice when gated by IgM. The developmental stage of the B cells is indicated below. (**G**) Percentage of MHC-II-positive developmental B cells in WT ($n = 7$), Het ($n = 5$) and KO ($n = 5$) mice when gated by IgD in (**E**). For (**A–G**) data represents mean ± SD. p values were calculated by two-way ANOVA with Sidak correction for multiple comparisons; $^{**}p < 0.01$, $^{***}p < 0.001$. p values for the percentage MHC-II cells comparisons are as follows: D (pre-B): WT vs Het: $p = 0.001$, WT vs KO: $p = 3.300\text{E-}14$, Het vs KO: $p = 1.238\text{E-}06$; E (immature): WT vs KO: $p = 1.223\text{E-}11$, Het vs KO: $p = 1.598\text{E-}06$. (**H**) Representative flow cytometry analysis of MHC-II-positive cells in D (pre-B), E (immature) and F (mature) subpopulations from WT, Het and KO littermates when gated by IgD. (**I**) Relative mRNA expression of total (pan-)CIITA, the three promoter-specific transcripts and ZBTB48 mRNA in D (pre-B), E (immature), and F (mature) subpopulations from WT mice. Data represents mean ± SEM. Data points with gray circles are imputed due to lack of detectable Ct values. The $\log_2$ fold change is calculated relative to samples with imputed values. p values were calculated by multiple t tests with Holm-Sidak correction for multiple comparisons ($n = 3$); $^{*}p < 0.05$, $^{**}p < 0.01$, $^{***}p < 0.001$. For D vs F: pan-CIITA: $p = 0.008$, CIITA pIII: $p = 0.007$, CIITA pIV: $p = 6.100\text{E-}05$; For D vs E: CIITA pIV: $p = 0.05$.

 © The Author(s)

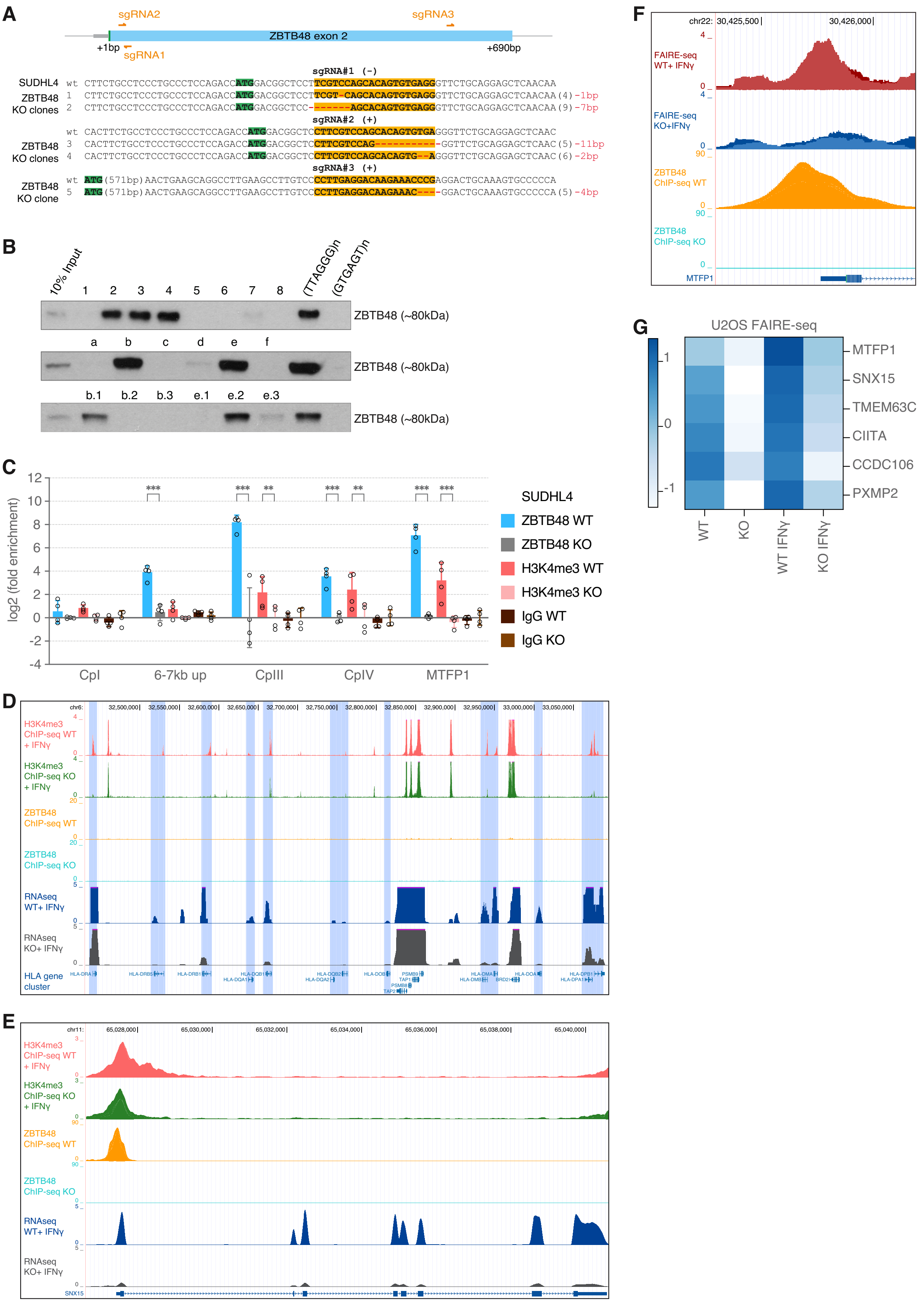
A
sgRNA2
sgRNA3
ZBTB48 exon 2
+1bp
sgRNA1
+690bp
sgRNA#1 (-)
SUDHL4 wt CTTCTGCCTCCCTGCCCTCCAGACCATGGACGGCTCCTTCGTCCAGCACAGTGTGAGGGTTCTGCAGGAGCTCAACAA
ZBTB48 KO clones 1 CTTCTGCCTCCCTGCCCTCCAGACCATGGACGGCTCCTTCGT-CAGCACAGTGTGAGGGTTCTGCAGGAGCTCAACAA(4)-1bp
2 CTTCTGCCTCCCTGCCCTCCAGACCATGGACGGCTCC-------AGCACAGTGTGAGGGTTCTGCAGGAGCTCAACAA(9)-7bp
sgRNA#2 (+)
wt CACTTCTGCCTCCCTGCCCTCCAGACCATGGACGGCTCCTTCGTCCAGCACAGTGTGAGGGTTCTGCAGGAGCTCAAC
ZBTB48 KO clones 3 CACTTCTGCCTCCCTGCCCTCCAGACCATGGACGGCTCCTTCGTCCAG-----------GGTTCTGCAGGAGCTCAAC(5)-11bp
4 CACTTCTGCCTCCCTGCCCTCCAGACCATGGACGGCTCCTTCGTCCAGCACAGTG--AGGGTTCTGCAGGAGCTCAAC(6)-2bp
sgRNA#3 (+)
wt ATG(571bp)AACTGAAGCAGGCCTTGAAGCCTTGTCCCCTTGAGGACAAGAAACCCGAGGACTGCAAAGTGCCCCCA
ZBTB48 KO clone 5 ATG(571bp)AACTGAAGCAGGCCTTGAAGCCTTGTCCCCTTGAGGACAAGAAAC----GGACTGCAAAGTGCCCCCA(5)-4bp
B
10% Input
1 2 3 4 5 6 7 8
(TTAGGG)n
(GTGAGT)n
ZBTB48 (~80kDa)
a b c d e f
ZBTB48 (~80kDa)
b.1 b.2 b.3 e.1 e.2 e.3
ZBTB48 (~80kDa)
C
log2 (fold enrichment)
12
10
8
6
4
2
0
-2
-4
***
***
**
***
**
***
***
CpI
6-7kb up
CpIII
CpIV
MTFP1
SUDHL4
ZBTB48 WT
ZBTB48 KO
H3K4me3 WT
H3K4me3 KO
IgG WT
IgG KO
D
chr6:
32,500,000
32,550,000
32,600,000
32,650,000
32,700,000
32,750,000
32,800,000
32,850,000
32,900,000
32,950,000
33,000,000
33,050,000
H3K4me3 ChIP-seq WT + IFNγ
H3K4me3 ChIP-seq KO + IFNγ
ZBTB48 ChIP-seq WT
ZBTB48 ChIP-seq KO
RNAseq WT+ IFNγ
RNAseq KO+ IFNγ
HLA gene cluster
HLA-DRA
HLA-DRB5
HLA-DRB1
HLA-DQB1
HLA-DQA1
HLA-DQB2
HLA-DQA2
HLA-DOB
PSMB9
TAP1
PSMB8
TAP2
HLA-DMA
HLA-DMB
BRD2
HLA-DOA
HLA-DPB1
HLA-DPA1
E
chr11:
65,028,000
65,030,000
65,032,000
65,034,000
65,036,000
65,038,000
65,040,000
H3K4me3 ChIP-seq WT + IFNγ
H3K4me3 ChIP-seq KO + IFNγ
ZBTB48 ChIP-seq WT
ZBTB48 ChIP-seq KO
RNAseq WT+ IFNγ
RNAseq KO+ IFNγ
SNX15
F
chr22:
30,425,500
30,426,000
FAIRE-seq WT+ IFNγ
FAIRE-seq KO+IFNγ
ZBTB48 ChIP-seq WT
ZBTB48 ChIP-seq KO
MTFP1
G
U2OS FAIRE-seq
1
0
-1
MTFP1
SNX15
TMEM63C
CIITA
CCDC106
PXMP2
WT
KO
WT IFNγ
KO IFNγ

Figure EV5. ZBTB48 also binds to CIITA pIII and regulates H3K4me3 levels in the constitutively expressing DLBCL cell line SUDHL4.

(**A**) Schematic of sgRNA design for the generation of ZBTB48 KO clones in SUDHL4. 5 different KO clones were identified and genotyped as shown. The start codon is shown in green and the sgRNA binding sites are highlighted in bold and yellow. (**B**) Western blot of DNA pull-down assay using SUDHL4 nuclear extracts demonstrates ZBTB48 binding at two sites within pIII. Telomeric (TTAGGG) and scramble control (GTGAGT) sequence are used as positive and negative controls, respectively.
(**C**) ZBTB48 and H3K4me3 ChIP reactions from four independent SUDHL4 WT and ZBTB48 KO clones analyzed by qPCR for CIITA pI, pII (6–7 kb up), pIII, pIV. The MTFP1 promoter is used as a positive control. The data was normalized to IgG and gene desert region and fold change was calculated relative to the average of WT clones. Data represents mean ± SD. p values were calculated by one-way ANOVA with Holm-Sidak correction for multiple comparisons ($n = 4$); *$p < 0.05$, **$p < 0.01$, ***$p < 0.001$. p values for WT vs KO comparisons: for ZBTB48 enrichment: 6–7 kb up: $p = 4.397\text{E-}06$, CpIII: $p < 1\text{E-}15$, CpIV: $p = 2.986\text{E-}06$, MTFP1: $p < 1\text{E-}15$; for H3K4me3 enrichment: CpIII: $p = 0.009$, CpIV: $p = 0.003$, MTFP1: $p = 3.162\text{E-}06$. (**D**) ZBTB48 and H3K4me3 ChIP-seq tracks with concomitant RNA-seq tracks in U2OS WT and ZBTB48 KO clones +/− IFNγ treatment (250 U/ml IFNγ for 48 h) covering the MHC-II gene cluster. The location of individual HLA genes is highlighted in light blue across tracks. (**E**) ChIP-seq tracks depicting ZBTB48 at the SNX15 promoter in U2OS WT cells but not in the KO cells with concomitant loss of RNA expression as seen in the RNA-seq tracks. In contrast, the H3K4me3 peak is present at the SNX15 promoter in both U2OS WT and ZBTB48 KO clones. (**F**) FAIRE-seq tracks depicting open chromatin at the MTFP1 promoter in two independent IFNγ-induced U2OS WT cells but not in the KO cells. The ZBTB48 ChIP-seq tracks highlight the ZBTB48 binding region within the MTFP1 promoter. (**G**) Heatmap of FAIRE-seq profiles depicting the relative chromatin accessibility at ZBTB48 bound promoter in U2OS WT vs ZBTB48 KO cells +/− IFNγ treatment (250 U/ml IFNγ for 48 h). Intensity values are based on Z-scores of log2 rpkm values across samples. Source data are available online for this figure.

 © The Author(s)
